# When Saying Sorry May Not Help: The Impact of Apologies on Social Rejections

**DOI:** 10.3389/fpsyg.2017.01375

**Published:** 2017-08-11

**Authors:** Gili Freedman, Erin M. Burgoon, Jason D. Ferrell, James W. Pennebaker, Jennifer S. Beer

**Affiliations:** ^1^Dartmouth College, Hanover NH, United States; ^2^Department of Psychology, University of Texas at Austin, Austin TX, United States

**Keywords:** social rejection, hurt feelings, apologies, forgiveness, language

## Abstract

If you have to socially reject someone, will it help to apologize? Social rejection is a painful emotional experience for targets, yet research has been silent on recommendations for rejectors. Across three sets of studies, apologies increased hurt feelings and the need to express forgiveness but did not increase feelings of forgiveness. The investigation of hurt feelings arising from a social rejection is challenging because previous research has shown that participants are reluctant to admit they felt hurt by the rejection. The present research addressed the self-report issue in two ways. First, participants rated how much social rejections would hurt someone’s feelings as a function of whether an apology was included across various social rejection scenarios (Studies 1a–e). Second, aggressive behavior was measured in response to face-to-face social rejections that were manipulated to include or exclude apologies (Studies 2a–c). More specifically, Studies 1a–e (*N* = 1096) found that although individuals sometimes use apologies in social rejections, social rejections with apologies are associated with higher levels of explicit hurt feelings. Studies 2a–c (*N* = 355) manipulated the presence of an apology in face-to-face social rejections and found that social rejections with apologies cause more aggressive behavior. As in previous research, participants are reluctant to admit to feeling hurt. Finally, Study 3 (*N* = 426) found that in response to social rejections with apologies, individuals feel more compelled to express forgiveness despite not actually feeling more forgiveness. Implications for the role of language in social rejections are discussed.

## Introduction

Imagine you find out that your friend gets lunch every Friday with your mutual coworkers. You ask to join, but your friend declines your request. Is there anything about the way your friend phrased the declination that would make you feel more or less hurt? More broadly, what insights can psychological science provide for people in the unenviable position of having to socially reject someone? Social rejection occurs when a rejector (i.e., perpetrator) denies the target (i.e., recipient) a requested social interaction (Leary, 1990; Williams, 1997; Molden et al., 2009). Social rejection can occur in a wide range of domains from romantic interactions (turning down a date) to interactions with a friend (telling a friend that you do not want him or her to join your lunch group) and even interactions with a stranger (telling a new classmate you do not want to work on a project together). Although there has been a great deal of research focused on social rejection, the research has almost exclusively focused on the targets of social rejection rather than those who carry out the rejection and how they do it. In fact, a key issue for would be rejectors is that of scriptlessness: there are no good scripts for engaging in rejection (Baumeister et al., 1993), and social psychological research has not yet uncovered the impact of different language choices on the target of rejection.

Ultimately, if one goal of conducting research on social rejection is to find ways to minimize its negative emotional effects, then psychologists need to investigate the full phenomenon of social rejection including the goal of providing empirically based advice for rejectors. The present set of studies examines not only what language choices rejectors make, but also how those choices impact targets. Specifically, the present research considers how apologies impact the perceived emotional impact of rejection. Whether apologies are helpful or harmful in the context of social rejections can provide insight about either (a) language that helps to mitigate the negative impact or (b) language that should be avoided to mitigate the negative impact.

The value of investigations aimed at understanding how rejectors can mitigate the damage of rejection is clear in light of the ample evidence that targets of social rejection suffer from a wide range of negative consequences. Social rejection is not only one of the main causes of hurt feelings (Leary et al., 1998; Feeney, 2004), it also can have physical consequences. For example, after people are socially rejected, they can experience increased physical and emotional pain (e.g., Leary, 1990; Leary et al., 1998; MacDonald and Leary, 2005; Eisenberger et al., 2006; Tang and Richardson, 2013) or physical and emotional numbness (e.g., Twenge et al., 2003, 2007; DeWall and Baumeister, 2006). The negative consequences are so strong that people will pay to avoid being socially rejected (van Beest and Williams, 2006). Furthermore, targets experience negative outcomes even when the source of the social rejection is a strongly disliked group (Gonsalkorale and Williams, 2007) or an inanimate object (Zadro et al., 2004).

Despite the great deal known about how targets fare, much less is known about the rejectors (Zadro and Gonsalkorale, 2014; Freedman et al., 2016). Some rejectors use social rejection as a method of social control and punishment (Wesselmann et al., 2013; Gooley et al., 2015; Wirth et al., 2015). However, social rejection is not always punitive: there are times when people cannot accept all invitations or wish to avoid a social encounter but are not trying to injure the target (Freedman et al., 2016). In cases where punishment is not the goal, rejectors often find it difficult and unpleasant to engage in social rejection (Folkes, 1982; Baumeister et al., 1993; Kets de Vries and Balazs, 1997; Ciarocco et al., 2001; Clair and Dufresne, 2004; Grunberg et al., 2006; Chen et al., 2014; however, see Zadro et al., 2005 for evidence that ostracism may be less difficult if you have peers also engaging in ostracism) and want to protect targets’ feelings (Goffman, 1967; Folkes, 1982; Baumeister et al., 1993; Besson et al., 1998; Tom Tong and Walther, 2010). But almost nothing is known about how rejectors can protect targets’ feelings.

## Apologies in Social Rejections

One untested question about how rejectors can achieve their goal of minimizing targets’ hurt feelings is whether they should include or avoid apologies when communicating social rejection. That is, it is important to understand whether apologizing is helpful or harmful in rejections because apologies are a common strategy for repairing broken bonds (Darby and Schlenker, 1982; Scher and Darley, 1997; Hodgins and Liebeskind, 2003; Eaton and Struthers, 2006; Hannon et al., 2010). Since apologies often work in the context of other social transgressions, individuals in the position to reject someone might consider using them within the rejection. Apologies can function in two ways to make forgiveness more likely: they can reduce the negative impression of the transgressor (Darby and Schlenker, 1982) and they can make the victim feel less angry and behave less aggressively (Ohbuchi et al., 1989).

However, apologies can also lead to negative consequences for both the speaker and the listener (Brown and Levinson, 1987; Freedman et al., 2016). Both Politeness Theory (Brown and Levinson, 1987) and the Responsive Theory of Social Exclusion (Freedman et al., 2016) suggest that apologies in social rejections will not decrease targets’ hurt feelings. Instead, apologies may backfire within a social rejection because they may make targets feel compelled to express forgiveness without actually making targets feel forgiveness and may make the target feel the rejector is not sincere. Social norms dictate that we forgive someone if they apologize; therefore, targets are put in a position where they are expected to forgive the rejection (Brown and Levinson, 1987) even if they do not believe the apology is sincere (Freedman et al., 2016).

## The Present Research

The present article reports a set of studies on the language of rejection that address two research questions. First, how likely are individuals to apologize in the context of social rejection? Second, how successful are apologies for mitigating targets’ hurt feelings and increasing forgiveness for the rejection? Prior work on apologies indicates that apologies have the potential to either be harmful or helpful. Therefore, Study 1a was an exploratory study designed to assess the impact of apologies on perceptions of rejections. Based on the results of Study 1a, Studies 1b–d, 2, and 3 were designed to test the damaging effects of apologies. Furthermore, we predicted that some rejections would include apologies, as apologies are a common strategy for repairing social bonds (Darby and Schlenker, 1982; Hodgins and Liebeskind, 2003; Eaton and Struthers, 2006; Hannon et al., 2010). Study 1a examined how likely participants are to include apologies in their rejections and whether rejections with apologies are more hurtful or helpful than rejections without apologies. Studies 1b–1d were replications of Study 1a in various social rejection scenarios. Study 1e was a meta-analysis of Studies 1a–d. Studies 2a and 2b manipulated the presence of an apology and examined the impact of rejections with apologies on explicit hurt feelings and aggressive behavior, and Study 2c was a meta-analysis of Studies 2a and 2b. Study 3 examined whether rejections with apologies garner a sense that forgiveness should be granted even if not felt and the role of sincerity in perceptions of rejections with apologies.

## Studies 1A–E

### Studies 1a–d

Studies 1a–d measured what participants spontaneously chose to include in their rejections in response to specific requests for social inclusion and how their choices affect the feelings of the requestor (i.e., recipient of social rejection). These rejections were then coded for the presence or absence of apologies. A separate group of raters rated how hurt they would feel if they received the rejections. All procedures for Studies 1a-e were approved by the University of Texas at Austin Institutional Review Board.

#### Method

Each of Studies 1a–d shared the same methodology but involved (a) unique participants, (b) unique social rejection situations, and (c) unique independent raters to investigate how the language of a social rejection makes a rejection better or worse. A general procedural description is provided for all studies and complemented by specific aspects of individual studies. Study 1a was the original exploratory study and Studies 1b-1d were run as confirmatory conceptual replications of Study 1a (Koole and Lakens, 2012; Simons et al., 2014).

##### Participants

As part of a larger study, participants were recruited from an Introductory Psychology course, local eating venues, outdoor events, festivals, and various locations on a university campus. Originally, 1304 participants responded to the survey, and of those 208 were excluded. The percent exclusion per study ranged from 13 to 28%. Specifically, 81 participants (16.84%) were excluded from Study 1a, 28 participants (13.02%) were excluded from Study 1b, 56 participants (28.72%) were excluded from Study 1c, and 43 participants (20.98%) were excluded from Study 1d.

Participants were excluded for one of four reasons across the four studies. In each study, participants were asked to write a response that they thought would be a “good way of saying no” in a scenario that called for social rejection. For example, in Study 1b, the instructions were as follows: “Write what you think would be a good way of telling Taylor that you do not want to go on a date. Please write exactly what you would say to Taylor.” Therefore, the highest number of exclusions came from participants not following instructions. Specifically, 162 participants were excluded for not writing a direct rejection, but instead writing the principle behind what they “would” say (e.g., “I would have to blame it on the person specifically that did not want him to attend the lunch”). Nineteen participants were excluded for not writing an actual response, but instead summarizing their state of mind (e.g., “Being patient and calm”) or saying they do not know what to say (e.g., “I don’t have a good answer to that question”). Fourteen participants were excluded for providing a response that did not make sense given the question (e.g., “999”). Finally, thirteen participants were excluded for not taking the task seriously (e.g., “You seem like a good person, Taylor, but I just died. Seriously. RIP Me.”). The raters did not see any of the excluded responses and there were no ratings for those responses. To avoid biasing the raters, cases that were excluded were never provided to them. That is, exclusions were performed before any rating or analysis took place. Across the four studies, 1096 participants provided useable responses to the experimental procedures and 1058 of those provided demographic information (631 females; *M*_age_ = 24.40 years, *SD* = 9.40).

The findings from Study 1a were investigated for their generalization to different populations (and different rejection scenarios, see below) in Studies 1b–d. Demographic information from each sample is provided and planned tests were conducted using a meta-analytic approach. Study 1a consisted of 481 participants recruited only from an Introductory Psychology course: 277 females, *M*_age_ = 19.04, *SD* = 2.20. Power was not calculated *a priori* for Study 1a as it used a convenience sample. Studies 1b–d included more diverse samples (Study 1b: *N* = 215, 78.6% community sample, 21.4% university sample, 103 females, *M*_age_ = 27.33, *SD* = 9.86; Study 1c: *N* = 195, 76.9% community sample, 23.1% university sample, 111 females, *M*_age_ = 30.28, *SD* = 11.52; Study 1d: *N* = 205, 72.7% community sample, 27.3% university sample, 113 females, *M*_age_ = 28.73, *SD* = 10.51). The target *N* for each of Studies 1b–d was 200 participants, which was based on a power analysis from the effect size from Study 1a (*d* = 0.47) with 90% power. The effect size from Study 1a was used, as there is no prior research on the relationship between apologies and perceptions of rejections. Therefore, the effect size from Study 1a was the most relevant one for Studies 1b–d.

##### Procedure

In each study, the procedure consisted of three distinct components. First, participants were presented with one of four social rejection scenarios (each study was a different scenario, see below) and asked to write a good rejection. Second, the rejections were coded for apologies. Finally, teams of independent raters evaluated the rejections for their impact on hurt and accepted feelings. These three sets of data sources were then analyzed to investigate how apologies were related to hurt feelings of the target.

*Rejectors’ responses*. In each study, participants were asked to write a response that they thought would be a “good way of saying no” in a scenario that called for social rejection. No further definition of “good way of saying no” was given to avoid biasing responses. For example, in Study 1b, the instructions were as follows:

*Write what you think would be a* ***good way*** *of telling Taylor that you do not want to go on a date. Please write exactly what you would say to Taylor.*

The target in each scenario was identified as “Taylor” (i.e., a gender-neutral name). The scenarios varied on characteristics such as whether the desire to reject was external (e.g., pressure from a group) or internal (e.g., lack of interest in a date) and how expected the rejection may have been (e.g., rejection is common in online dating but perhaps less common from a friend). The scenarios were selected to represent a breadth of common rejection experiences. Social rejection occurs across a range of interpersonal relationships (romantic, friend, coworker, acquaintance; Blackhart et al., 2009), and one goal of Study 1 was to examine the role of apologies in rejections across these different domains.

Study 1a (Lunch). Participants imagined that Taylor would like to attend a weekly lunch group, yet the friends already in the lunch group do not want to include anyone else:

At your job, you go out for lunch with the same four colleagues, Pat, Emily, Michael, and Jennifer, every Friday. Friday is the only day all of you have time to go out and all of you make it a point to attend the lunch. You really enjoy their company and your weekly lunch with them. Pat, Emily, Michael, and Jennifer have made it clear that they are not open to including more people at lunch. You also like your colleague Taylor. One day, you get an email from Taylor. Taylor overheard someone talking about your Friday lunches with Pat, Emily, Michael, and Jennifer, and Taylor wants to join. Taylor’s email to you: Hey! I heard that you, Pat, Emily, Michael, and Jennifer all get lunch together on Fridays, and I was wondering if I could join you guys? Let me know! –Taylor

Study 1b (Romantic). Participants imagined that Taylor has asked for a date via an online dating site:

Imagine that you are a member of an online dating site. You have exchanged emails with someone named Taylor. One day Taylor emails you asking you out on a date. You do not want to go on a date with Taylor.

Study 1c (Party). Participants imagine that Taylor requests to get together after meeting the participant at a party:

Imagine that you are at a party at a friend’s place. At this party you meet someone named Taylor. The week after the party, it seems that Taylor would like to become friends and keeps suggesting times to hang out. You have no interest in having a close friendship with Taylor. You finally decide that you need to tell Taylor that you don’t want to hang out one-on-one.

Study 1d (Roommate). Participants imagine that Taylor is the participant’s roommate and asks to live together again:

Imagine that you have been living with a roommate named Taylor for almost a year. You have decided that there is no way you will live with Taylor again, but Taylor doesn’t know this. One day Taylor asks you if you want to continue living together.

*Apologies*. The presence of an Apology was calculated objectively by counting the number of cases with any form of the words “sorry” or “apology.”

*Responses to rejection*. Across the four studies, raters blind to hypotheses were randomly assigned to imagine themselves in Taylor’s position in one of the four scenarios. There were five raters for Study 1a, and three raters for each of the other three studies. Prior research on reliability among raters indicates that, with 100 or more language coding samples, using three raters or fewer is acceptable (Shoukri et al., 2004), and the use of two or three raters is common in research on rejection that involves coding language (e.g., Sommer et al., 2001; Leary et al., 2003; Smith and Williams, 2003). The raters evaluated each participant’s rejection (7-point Likert scale ranging from *not at all* to *very*) for Rejected Feelings (2 items: how hurt and accepted [reverse-scored] they would feel if they received that rejection). Each study involved a unique group of raters and demonstrated acceptable reliability (mean α = 0.84, range = 0.73-0.97).

#### Results

Consistent with our hypotheses, apologies were a feature of some of the social rejections written in response to hypothetical scenarios. Across the four studies, 39% of the rejections contained an apology (Lunch: 52.3%; Romantic: 29.3%; Party: 34.9%; Roommate: 22.1%). Also consistent with our hypotheses (e.g., Brown and Levinson, 1987; Freedman et al., 2016), the use of apologies in social rejections increased hurt feelings.

In the Lunch scenario (1a), social rejections with apologies were significantly associated with more hurt feelings (*M* = 3.92, *SD* = 0.62) than social rejections without apologies (*M* = 3.62, *SD* = 0.63; *t*(479) = 5.20, *p* < 0.001, *d* = 0.47, 95% CI [0.19, 0.41]). In the Romantic scenario (Study 1b), there was no association of apologies with hurt feelings: *t*(213) = 0.501, *p* = 0.617, *ns*, 95% CI [-0.20, 0.34]. In the Party scenario (Study 1c), social rejections with apologies were marginally associated with more hurt feelings (*M* = 4.65, *SD* = 0.84) than social rejections without apologies (*M* = 4.37, *SD* = 1.17; *t*(193) = 1.80, *p* = 0.074, *d* = 0.27, 95% CI [-0.03, 0.60]). Finally, in the Roommate scenario (Study 1d), social rejections with apologies were significantly associated with more hurt feelings (*M* = 4.82, *SD* = 0.66) than social rejections without apologies (*M* = 4.51, *SD* = 0.81; *t*(202) = 2.39, *p* = 0.018, *d* = 0.40, 95% CI [0.05, 0.57]).

#### Discussion

Studies 1a–d found that apologies were included in an average of 39% of the social rejections written in response to hypothetical scenarios, but the inclusion of apologies ranged from 22 to 52% across the individual studies. Furthermore, these studies suggest that rejections with apologies did not lead to decreased hurt feelings and were in fact generally associated with more hurt feelings. However, one concern with Studies 1a–d was the differing exclusion rate across the studies. In particular, Study 1c had a higher exclusion rate of about 28%. It is possible that the scenario of meeting someone at a party and having that person want to continue to interact later was one that the participants had not encountered before leading to confusion and answers that had to be excluded. In order to examine if the association between apologies and hurt feelings is robust across the different studies despite the differing levels of exclusion, Study 1e was conducted as a meta-analysis of Studies 1a–d.

### Study 1e

Study 1e used the data from Studies 1a–d to perform a meta-analysis. As a field, social psychology has raised recent concerns about adequate sample size, power, and reproducibility, and critiques of the statistics within psychological science have stressed the importance of using meta-analytic techniques even across a few studies (Maxwell, 2004; Ioannidis, 2005; Pashler and Wagenmakers, 2012; Cumming, 2014; Fraley and Vazire, 2014). A meta-analysis allows for a more reliable estimation of the overall effect size.

#### Methods

A fixed-effects meta-analysis of the effects size of the effect of apologies on hurt feelings in Studies 1a–d was performed using the metafor package in R (Viechtbauer, 2010).

#### Results

**FIGURE 1:**
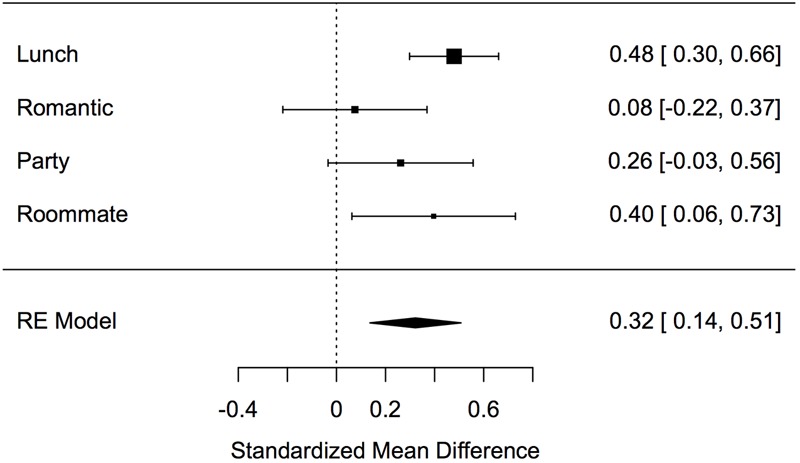
Effect sizes of Studies 1a–d.

Results indicate a significant effect of apologies on hurt feelings, *p* < 0.001, *d* = 0.35, 95% CI of *d* [0.23, 0.48] (see **Figure 1**). Further, an analysis of heterogeneity of the studies reveals no significant difference between Experiments 1a–d, *Q*(3) = 5.5464, *p* = 0.14, *ns*.

#### Discussion

Study 1e reinforces the association between apologies and hurt feelings. The meta-analysis provides an overarching estimate of the effect size associated with conclusions drawn from Studies 1a–d.

## Studies 2A–C

Studies 2a and 2b expand upon the association between apologies and hurt feelings by investigating them in a face-to-face interaction. In order to combat the self-presentational issues found in previous research on social rejection (Bernstein et al., 2013), we employ a standardized paradigm to test whether the words “I’m sorry” elicit responses consistent with hurt feelings. Whereas Study 1 had a third party rate hurt feelings, previous research has found that sometimes direct targets of rejection are reluctant to admit to hurt feelings but then behave in ways that suggest that they are in fact hurt (Bernstein et al., 2013). Study 2a includes an explicit measure of hurt feelings and a measure of aggression, which may be considered an implicit indicator of hurt feelings (DeWall et al., 2010); 2b is a replication of Study 2a but only includes the aggression measure. All procedures for Studies 2a–c were approved by the University of Texas at Austin Institutional Review Board.

### Study 2a

#### Method

##### Participants

We planned our sample size by averaging the effect sizes from two studies that report the impact of rejection on hot sauce administration (Ayduk et al., 2008; Wesselmann et al., 2010). These two studies were chosen because both use laboratory-based rejection in which the participant is told that he or she is not wanted as a partner (Ayduk et al., 2008) or group member (Wesselmann et al., 2010), which was the most similar paradigm to the one used in the present study. Specifically, the present study involves a confederate speaking to a participant prior to a brief social rejection statement. In the group rejection paradigm (Wesselmann et al., 2010), a confederate speaks to the participant prior to the rejection and in the partner paradigm (Ayduk et al., 2008), there is a brief statement about how the participant is rejected. Therefore, we calculated an average for the two effect sizes for an expected effect size for the present study. Thus, based on a power analysis with *d* = 0.46 and 80% power for an independent samples *t*-test, we recruited 151 participants from Introductory Psychology classes to take part in the experiment. Fourteen participants were excluded for suspicion of the confederate, one participant was excluded for not following instructions, and one participant was excluded for knowing the purpose of the experiment; an exclusion rate consistent with other deception studies which typically range between 5 and 25% (Stricker et al., 1969; Gardner et al., 2000; Baumeister et al., 2005). This left a final sample of 135 (97 females; *M*_age_ = 18.90 years, *SD* = 1.74).

##### Procedure

The presence of an apology was manipulated during a face-to-face social rejection and its effect on hot sauce allocation was examined. Reactions to rejections with and without apologies were assessed using both an explicit measure and an aggression measure. The aggression measure was used to address people’s reluctance to admit their own hurt feelings in the face of social rejection (Bernstein et al., 2013). The aggression task (hot sauce administration) has often been used in experiments on the negative consequences of social rejection (Lieberman et al., 1999; Klinesmith et al., 2006; Ayduk et al., 2008; Wesselmann et al., 2010) including those interested in measuring hurt feelings (e.g., DeWall et al., 2010).

In each session, a participant and a confederate were told they would perform several tasks. First, they each completed a taste preference form, which is the first phase of the hot sauce allocation task (Lieberman et al., 1999); the confederate always indicated a strong aversion to spicy foods. The experimenter stepped out of the room. During this time, the confederate engaged the participant in a friendly, standardized 1-min conversation while waiting for the experimenter to return. In this conversation, the confederate asked the participant what introductory psychology class he or she was in and whether he or she had done any other psychology studies yet. The confederate always indicated that he or she was in the “big” introductory psychology class (i.e., one with over 1000 students) and that he or she had only done an online study so far. The experimenter then returned and used a coin flip procedure designed to ensure that the confederate would be asked to decide whether the confederate and participant would work together (or alone) on the next task. At each session, the confederate (who was blind to hypotheses) was randomly assigned to verbally reject the participant using the same words with the exception that sometimes they included an apology (“No. I’m sorry. I don’t want to work with you.”), and sometimes they did not (“No. I don’t want to work with you.”). Experimenters were unable to be blind to condition because they witnessed the confederate state an apology (or not); however, they were blind to hypotheses about the apology manipulation. After completing a filler task (a word search) and the explicit measure of hurt feelings (on a 1–7 scale, how hurt do you feel), participants completed the final stage of the hot sauce allocation task. They were given the confederate’s taste preference form indicating his/her dislike of spicy foods and asked to allocate hot sauce for the confederate to taste. The participant was informed that the confederate would have to consume the entirety of the sample and that the experimenter would remain blind to the amount. Participants were then debriefed. Hot sauce was weighed in grams using a digital scale.

Confederates trained extensively prior to the experiment to ensure that they performed identically across the two conditions. Specifically, confederates were instructed and trained to maintain the same tone of voice and non-verbal expressions in both the Apology and No Apology conditions. For example, as part of the training, confederates had their voices recorded during trials of both conditions. Those recordings were played back to them so they could adjust their tones of voice to make sure that the only difference between the two conditions was the presence or absence of “I’m sorry.”

#### Results

Consistent with our hypothesis, participants allocated more hot sauce when they received an apology (*M* = 5.82 g, *SD* = 9.15 g) than when they did not (*M* = 3.19, *SD* = 5.84; *t*(133) = 2.00, *p* = 0.047, *d* = 0.35, 95% CI of mean difference [0.04, 5.2]). The explicit measure of hurt feelings showed no effect of apology [*t*(149) = 1.10, *p* = 0.27]. The means for the explicit measure of hurt feelings in both conditions indicated a likely floor effect (Apology *M* = 1.88, *SD* = 1.20; No Apology *M* = 1.67, *SD* = 1.08), which is congruent with the research showing that people are reluctant to admit hurt feelings after social rejection due to self-presentation concerns (Bernstein et al., 2013).

To test for confederate effects, a 2 (Apology vs. No Apology) × 7 (Confederates) ANOVA was used to analyze the data. Confederate identity did not moderate the effect of apology on hot sauce [*F*(6,121) = 0.85, *p* = 0.53, *ns*]. Furthermore, an analysis on whether confederate gender was a match or mismatch with participant gender was conducted to check for gender effects on the outcome. A 2 (Apology vs. No Apology) × 2 (Match vs. Mismatch) ANOVA did not find evidence for a moderation by gender match: *F*(1,131) = 0.04, *p* = 0.84. Furthermore, there was no evidence of an interaction of Apology and Gender of participant [*F*(1,130) = 0.11, *p* = 0.75] or a main effect of gender on hot sauce allocation [*F*(1,130) = 0.25, *p* = 0.62].

#### Discussion

As predicted, rejections that contained the words “I’m sorry” led to worse outcomes than rejections without apologies; participants allocated more hot sauce. The participants believed the confederate did not like spicy food and, therefore, the greater allocation likely reflected a hostile act designed to hurt the confederate (Lieberman et al., 1999). Study 2b was conducted to test for a replication of the effects from Study 2a.

### Study 2b

#### Method

Given the recent call for a greater focus on replication (e.g., Nosek et al., 2012; Funder et al., 2014), Study 2b was run as a replication testing the hypothesized effect on the aggression measure from Study 2a^1^.

##### Participants

Based on a power analysis using the effect size from Study 2a (*d* = 0.35) and 80% power we recruited 260 participants from Introductory Psychology classes for the experiment. Of the 260 participants, 13 participants were excluded for suspicion of the confederate, 2 were excluded due to experimenter or confederate error, and 6 were excluded for knowing the purpose of the experiment. Furthermore, three participants were excluded for extreme outlier scores on hot sauce allocation (greater than 7 standard deviations from the mean) for a total of 236 participants (145 females; *M*_age_ = 19.04 years, *SD* = 1.64). One of the outliers was in the Apology condition (245 g) and two were in the No Apology condition (189 and 196 g).

#### Results

Consistent with Study 2a, participants allocated marginally more hot sauce when they received an apology (*M* = 9.06 g, *SD* = 16.81 g) than when they did not (*M* = 5.81, *SD* = 10.81; *t*(233) = 1.76, *p* = 0.08, *d* = 0.23, 95% CI of mean difference [-0.39, 6.89]). Furthermore, these results were not moderated by confederate identity [*F*(9,211) = 0.78, *p* = 0.63], confederate-participant gender match [*F*(1,211) = 0.44, *p* = 0.51] or participant gender [*F*(1,211) = 0.50, *p* = 0.48].

#### Discussion

Study 2b replicated the finding from Study 2a that rejections that include the words “I’m sorry” cause people to allocate more hot sauce to their rejector. In both Studies 2a and 2b, the only difference between the conditions was the presence or absence of the words “I’m sorry.” All other differences were controlled.

### Study 2c

Due to current concerns in the field of social psychology about adequate sample size, power, and reproducibility (Maxwell, 2004; Ioannidis, 2005; Pashler and Wagenmakers, 2012; Fraley and Vazire, 2014), a meta-analysis on Studies 2a and 2b was conducted.

#### Method

As in Study 1e, a fixed-effects meta-analysis of Studies 2a and 2b was performed using the metafor package in R (Viechtbauer, 2010).

#### Results

Results indicate a significant effect of apologies on hot sauce allocation, *p* = 0.009, *d* = 0.27, 95% CI of *d* [0.07, 0.48]. Further, an analysis of heterogeneity of the studies reveals no significant difference between Experiments 2a and 2b, *Q*(1) = 0.2791, *p* = 0.60, *ns*, 95% CI of *d*3a [0.03, 0.49], 95% CI of *d*3b [0.0, 0.68].

#### Discussion

The meta-analysis of Studies 2a and 2b shows an effect of the words “I’m sorry” to increase hot sauce allocation, an aggression measure which has been associated with hurt feelings (DeWall et al., 2010), in face-to-face social rejections.

## Study 3

Study 3 extends Study 2 by both using a more controlled social rejection situation and testing how social rejections with and without apologies impact forgiveness. In Study 3, participants viewed videos of social rejections and indicated how the target of the social rejection would feel. We hypothesized that social rejections with apologies, but not social rejections without apologies, garner a sense that forgiveness should be granted even if not felt. Furthermore, Study 3 examined the role of sincerity in the context of social rejections with apologies (e.g., Freedman et al., 2016). That is, do people find apologies sincere when they are used in a social rejection? All procedures for Study 3 were approved by the University of Texas at Austin Institutional Review Board.

### Methods

#### Participants

Participants were recruited via Amazon’s Mechanical Turk in exchange for 25 cents. Four hundred ninety-three participants (249 female; *M_age_* = 33.3, *SD* = 11.2) completed the survey. The target sample size was 460 participants, which was based on a power analysis to be able to detect medium effects (*d* = 0.3) with 90% power. The effect size was based on a pilot study in the lab on apologies and forgiveness in rejections.

#### Procedure

In Study 3, participants viewed a video from the perspective of someone named Taylor. In this video, Taylor is rejected by a roommate named Jamie. All participants view Taylor reading a notification that the apartment is pre-leasing for next year. Taylor calls Jamie into the room and tells Jamie that they need to figure out housing. Taylor then says to Jamie, “It looks like we have to figure out housing for next year. Want to be roommates again?.” Jamie rejects Taylor at this point. In all cases, Jamie says “I’ve actually already found a different place. I’m rooming with Jesse.” In half of the videos, Jamie starts the social rejection by saying “I’m sorry.” Videos were shot with the full dialog (“I’m sorry. I’ve actually already found a different place. I’m rooming with Jesse.”), but the dialog was edited by cutting “I’m sorry” from half of the videos. Additionally, one of the original (i.e., pre-edited) videos was shot using two female actors and one of the original videos was shot using two male actors.

Participants indicated their demographics on an online survey. If they indicated that they were male, they were randomly assigned to one of the male videos. If they indicated that they were female, they were randomly assigned to one of the female videos.

After viewing the videos, participants were asked how sincere the apology in the video was with the option of “there was no apology in the video.” Participants were also asked how compelled would Taylor be to express forgiveness, how likely would Taylor be to express forgiveness, and how likely would Taylor be to feel forgiveness. As in previous experiments, participants were also asked how hurt they would feel and how accepted they would feel. Both questions were answered on a 1–7 scale.

### Results

#### Manipulation check

Out of the 493 participants, 426 (86%) correctly identified whether their video involved an apology (i.e., an acceptable rate for a manipulation check: Oppenheimer et al., 2009). For the following analyses, only those 426 participants’ responses were analyzed.

#### Forgiveness

As hypothesized, participants in the Apology condition were more likely to indicate that Taylor would feel compelled to express forgiveness (*M* = 4.27, *SD* = 1.71) than those in the No Apology condition (*M* = 3.53, *SD* = 1.90; *t*(423) = 4.24, *p* < 0.001, *d* = 0.41, 95% CI [0.40, 1.09]). In addition, participants in the Apology condition were more likely to indicate that Taylor would express forgiveness (*M* = 4.46, *SD* = 1.50) than those in the No Apology condition (*M* = 4.04, *SD* = 1.73; *t*(423) = 2.67, *p* = 0.008, *d* = 0.26, 95% CI [0.11, 0.73]). However, Study 3 found no significant difference in how likely Taylor would be to feel forgiveness in the Apology condition (*M* = 3.42, *SD* = 1.49) than the No apology condition (*M* = 3.40, *SD* = 1.58; *t*(423) = 0.19, *p* = 0.87; 95% CI [-0.27, 0.32]).

#### Hurt feelings

A Hurt Feelings score was computed by averaging how hurt would Taylor feel with a reverse-scored how accepted would Taylor feel. There was no difference in Hurt Feelings between Apology (*M* = 5.20, *SD* = 1.21) and No Apology (*M* = 5.21, *SD* = 1.13; *t*(424) = 0.11, *p* = 0.91, 95% CI [-0.24, 0.21]).

#### Sincerity

Rejections with apologies were perceived as less sincere (*M* = 3.29, *SD* = 1.44) than rejections without apologies (*M* = 4.10, *SD* = 1.59; *t*(424) = 5.51, *p* < 0.001, 95% CI [0.52, 1.10], *d* = 0.54).

### Discussion

Study 3 found that participants who receive a social rejection with an apology feel obligated to express forgiveness but do not feel significantly more forgiveness. Furthermore, Study 3 also found that social rejections with apologies were perceived as less sincere than social rejections without apologies. Study 3 provides further evidence that apologizing in the context of a social rejection can be problematic for both parties. That is, Study 3 showed that rejections with apologies are perceived more negatively, which can have ramifications both for the target (i.e., the target will be less likely to feel forgiveness) and for the rejector (i.e., the rejector is less likely to gain forgiveness).

## General Discussion

The devastating consequences of social rejection highlight the value in understanding ways in which to soften the blow. The current research addresses this issue by asking how the language of social rejection impacts the emotional damage for target. Together, the findings provide a first step in evidence-based advice for how to socially reject someone in a less damaging way. The current research has made broad strides by identifying word choices in social rejections and the emotional consequences of those word choices: rejectors now have some beginning guidelines of what to say and what not to say. The next generation of research will be important for elaborating the boundary conditions of these effects.

More broadly, the present research is an important step in moving the understanding of social rejection forward. Prior work on social rejection has not examined how language choices influence the outcomes of social rejection for either party. The present set of studies indicates that the negative effects of social rejection are unlikely to be ameliorated with a simple apology. The lack of a positive impact of apologies on perceptions of rejections may stem from the perceived intentionality of rejection. Although apologies are helpful in cases of unintentional transgressions (e.g., accidentally spilling your drink on someone), when individuals apologize for an intentional transgression (e.g., intentionally spilling your drink on someone), the apology backfires (Struthers et al., 2008). It is possible that social rejections are perceived as intentional and therefore an apology falls flat. That is, regardless of the constraints a rejector faces (e.g., two social events at the same time), a target of social rejection might think that the rejector could have chosen not to reject and that, therefore, engaging in rejection was an intentional act. Future research should consider whether there are certain types of rejection that are perceived as more or less intentional and if apologies are better received for the unintentional rejections.

The present research shows that it is not the case that any nicety will be effective in minimizing the emotional damage of social rejection. In fact, some social niceties may backfire. That is, although an average of 39% of Study 1 participants spontaneously included an apology in their attempt to craft a less emotionally damaging social rejection, all of the present studies indicated that apologies are unlikely to significantly decrease hurt feelings. The gap between lay intuitions and the effects of those beliefs may cause social rejections to be more emotionally unpleasant for both parties than is actually necessary.

Although apologies have benefits in other domains, their damaging impact in social rejection reinforces other findings on the interpersonal dynamics of social rejection. When a person receives an apology, social norms of politeness and scripts constrain their response options (Schank and Abelson, 1977; Brown and Levinson, 1987), and Study 3 supported this idea by suggesting that people who perceive apologies are more likely to express forgiveness but not more likely to feel forgiveness.

An important consideration for future research on the role of language in social rejection is considering what the rejectors’ goals are in the interaction. For example, rejectors may try to reduce targets’ hurt feelings, they may try to make themselves feel better, or they may just want the quickest solution (i.e., the most efficient form of rejection). In terms of apologies, rejectors may want to use them even if they do not make targets feel better. That is, it is possible that rejectors apologize to make themselves feel better rather than to make the target feel better. Although an ideal situation would involve language that satisfies both parties, it would be important to consider how well apologies act to alleviate the guilt of the rejectors. Furthermore, it is possible rejectors may choose to continue apologizing in social rejections if it reduces their guilt even if it will increase targets’ hurt feelings. Future research can examine how rejectors weigh the possible outcomes of using an apology to assess when they are likely to risk greater hurt feelings for the possibility of feeling better themselves.

### Limitations and Future Directions

The present studies are the first to examine how the language of social rejection, specifically the use of apologies, impacts emotional outcomes and have laid foundational groundwork for future research. They also highlight opportunities and challenges for future research that will continue to close the gap between the large body of research on the emotional impact of being rejected and the limited body of research on those who carry out the rejection. For example, the current research provides a springboard for understanding how time, attribution, and word length impact both targets and rejectors’ emotions.

An important challenge to consider for future research on rejection language and hurt feelings is the assessment of hurt feelings. Previous research has indicated that explicitly measuring hurt feelings can be problematic due to self-presentation concerns (Bernstein et al., 2013), and the present studies found continuing evidence of this assessment challenge. Although the present research did not manipulate or measure self-presentation concerns, the results across studies may indicate a potential self-presentation issue in measuring hurt feelings. Study 1 found evidence of the impact of apologies on explicitly measured hurt feelings perhaps because raters were able to avoid self-presentation concerns when they were rating multiple rejections. Furthermore, Study 2 found evidence that apologies increase aggression. Aggression is a well-documented response to rejection and one theory about rejection and aggression argues that the pain of rejection elicits aggressive responses (Leary et al., 2006). In other words, aggression in response to rejection may be an indicator of the rejected individual’s hurt feelings. Both Study 1a and Study 2a were internally replicated in the present set of studies providing evidence that there is a positive association between apologies and hurt feelings in the context of social rejection. However, Study 3 did not find an association between apologies and explicitly measured hurt feelings. It is possible that the vantage point of being the rejector, even through watching a video, may have led to presentational concerns that decreased willingness to indicate that the target may experience hurt feelings. It will therefore be paramount for future research on social rejection or more generally on hurt feelings to develop measures that are free from self-presentation concerns.

One question worthy of future consideration is how the timescale of social rejection impacts emotional outcomes. That is, the language in the present studies was examined in terms of one’s immediate reaction to the social rejection, and the results cannot speak to the impact of rejections of apologies on targets’ later feelings. It is possible that if there were more time between the social rejection and the reaction, the outcomes would change. For example, previous research has established that people who are especially sensitive to negative social cues experience a heightening of the negative effects of social rejection following a time delay (Zadro et al., 2006; Wirth and Williams, 2010; Perry et al., 2011). Future research can explore the ways in which time can alter emotional reactions to social rejection, for better and for worse.

Another important consideration for future research is how the co-occurrence and order language choices impact the consequences of a rejection. Future research should consider not only the impact apologies on social rejections, but also how apologies may interact with other features to impact hurt feelings and perceptions of the rejector. For example, in the business realm, rejection letter writers are advised to state the rejection at the beginning of the letter in order to avoid surprising the rejected job applicant (Locker, 1999). Previously, letter writers were cautioned to begin with a positive statement (i.e., a buffer), but research on buffers found that rejected applicants were more upset because they were then surprised by the rejection after reading something positive (Locker, 1999). Similarly, it is possible that social rejections may benefit from certain linguistic feature timings. For instance, is starting with an apology, indicating the rejection, and then providing some type of alternative to the denied request a less hurtful way of rejecting than beginning with a positive statement and ending with an apology?

Do target’s attributions for a rejection interact with language to shape emotional reaction? For example, are apologies highlighting a particular attribution? That is, if Tom rejects Anna’s request for a lunch date and apologizes, does Anna then interpret the rejection as something that was Tom’s fault simply because he offered an apology? Apologies are often associated with wrongdoing because of their usefulness in cases of interpersonal transgressions (e.g., Darby and Schlenker, 1982; Hodgins and Liebeskind, 2003; Eaton and Struthers, 2006; Hannon et al., 2010), and the association of apologies with wrongdoing may lead to negative attributions about the rejector. Future research can consider the attributional ramifications of the language findings arising from the current research to better understand the impact of language on emotional consequences of rejection.

Finally, it is important for future research to consider how non-verbal displays during a rejection may impact the way the rejection is received. For example, whether the rejector is making eye contact or avoiding the target may influence the target’s perception of the sincerity of parts of the rejection. An apology may come off as insincere if the rejector is unable to face the target directly. Furthermore, if the rejector is able to convey a sense that he or she is paying attention to the target and treating the target as an equal, the rejection may be perceived in a less negative light. Beyond eye contact, other aspects of non-verbal communication may be important for showing attention and respect including the way the rejector’s body is positioned and whether the rejector nods or provides other indications of listening to the target if the target responds to the rejection. It may in fact be especially important to engage in certain non-verbal displays during a rejection. People who have recently been rejected are particularly adept at decoding the sincerity of non-verbal displays (e.g., Duchenne vs. non-Duchenne smiles; Bernstein et al., 2008) and at remembering interpersonal events (Gardner et al., 2000).

## Conclusion

Taken together, the results indicate that not all social rejections are created equal, and it would behoove rejectors to carefully consider their language choices when constructing a social rejection to minimize emotional hurt. Although it often seems as though targets are the only ones suffering in the social rejection, it is difficult to reject someone (e.g., Folkes, 1982; Baumeister et al., 1993), and at times there are no other realistic options (e.g., if two friends each hold their wedding on the same day, you can only be in one place at a time). Social psychological science needs to continue to investigate the neglected half of social rejection, that is, the rejectors, to better understand how to mitigate its emotional consequences. Social rejection is a complex process and uncovering how language affects emotional experiences will bring us one step closer to understanding how to help both the target and the rejector.

## Ethics Statement

This study was carried out in accordance with the recommendations of the University of Texas at Austin Institutional Review Board with written or verbal informed consent from all subjects. All subjects gave written or verbal informed consent in accordance with the Declaration of Helsinki. The protocol was approved by the University of Texas at Austin Institutional Review Board. The University of Texas at Austin Institutional Review Board waived the requirement of written consent and approved verbal consent in its place for Studies 1b–d.

## Author Contributions

GF, EB, and JB developed the research questions. All authors assisted with designing the experiments. GF, EB, and JF ran the experiments. GF performed the data analysis. GF wrote the manuscript and all authors provided revisions of the manuscript.

## Conflict of Interest Statement

The authors declare that the research was conducted in the absence of any commercial or financial relationships that could be construed as a potential conflict of interest.

## Footnotes

1 Participants were also divided into Agency and No Agency creating four conditions: Apology, Agency; Apology, No Agency; No Apology, Agency; No Apology, No Agency. In Agency, participants were given a little bit of ostensible control over the role they would play in the next task whereas participants in No Agency were not given any control. Specifically, participants in the Agency condition were able to select a card from a face-down deck that would indicate which role they had (i.e., they did not know what they were choosing, they were merely engaging in the action of selecting) whereas the participants in No Agency had a card selected for them. There were no main effects or interactions with the Agency vs. No Agency condition (all *p* > 0.50). Therefore, the analyses focus on the Apology vs. No Apology manipulation.
